# Five-year serological and clinical evolution of chronic Chagas disease patients in Cochabamba, Bolivia

**DOI:** 10.1371/journal.pntd.0011498

**Published:** 2023-12-29

**Authors:** Jimy Pinto, Malia Skjefte, Julio Alonso-Padilla, Daniel Franz Lozano Beltran, Lilian Victoria Pinto, Aina Casellas, Mery Elena Arteaga Terrazas, Karen Alejandra Toledo Galindo, Roxana Challapa Quechover, María Escobar Caballero, Alejandra Perez Salinas, Mario Castellón Jimenez, Sergi Sanz, Joaquim Gascón, Faustino Torrico, María Jesús Pinazo

**Affiliations:** 1 Fundación Ciencia y Estudios Aplicados para el Desarrollo en Salud y Medio Ambiente (CEADES), Cochabamba, Bolivia; 2 Harvard TH Chan School of Public Health, Department of Global Health and Population, Boston, Massachusetts, United States of America; 3 Barcelona Institute for Global Health (ISGlobal), Hospital Clínic—University of Barcelona, Barcelona, Spain; 4 CIBER de Enfermedades Infecciosas, Instituto de Salud Carlos III (CIBERINFEC, ISCIII), Madrid, Spain; 5 Universidad Mayor de San Simón, Cochabamba, Bolivia; 6 Drugs for Neglected Diseases Initiative (DND*i*), Geneve, Switzerland; Universidade Federal de Minas Gerais, BRAZIL

## Abstract

**Background:**

Chagas disease, caused by the parasite *Trypanosoma cruzi*, is a neglected infectious disease that exerts the highest public health burden in the Americas. There are two anti-parasitic drugs approved for its treatment–benznidazole and nifurtimox—but the absence of biomarkers to early assess treatment efficacy hinders patients´ follow-up.

**Methodology/Principal findings:**

We conducted a longitudinal, observational study among a cohort of 106 chronically *T*. *cruzi-*infected patients in Cochabamba (Bolivia) who completed the recommended treatment of benznidazole. Participants were followed-up for five years, in which we collected clinical and serological data, including yearly electrocardiograms and optical density readouts from two ELISAs (total and recombinant antigens). Descriptive and statistical analyses were performed to understand trends in data, as well as the relationship between clinical symptoms and serological evolution after treatment. Our results showed that both ELISAs documented average declines up to year three and slight inclines for the following two years. The recorded clinical parameters indicated that most patients did not have any significant changes to their cardiac or digestive symptoms after treatment, at least in the timeframe under investigation, while a small percentage demonstrated either a regression or progression in symptoms. Only one participant met the “cure criterion” of a negative serological readout for both ELISAs by the final year.

**Conclusions/Significance:**

The study confirms that follow-up of benznidazole-treated *T*. *cruzi*-infected patients should be longer than five years to determine, with current tools, if they are cured. In terms of serological evolution, the single use of a total antigen ELISA might be a more reliable measure and suffice to address infection status, at least in the region of Bolivia where the study was done. Additional work is needed to develop a test-of-cure for an early assessment of drugs´ efficacy with the aim of improving case management protocols.

## Author summary

Chagas disease is a neglected parasitic disease prevalent throughout the Americas, with approximately seven million people infected. The highest infection rates have been identified in Bolivia. There, we followed for five years a cohort of chronically *T*. *cruzi-*infected patients attending the Platform for Integral Care of Chagas disease Patients in Cochabamba. With the aim to understand clinical and serological responses after completing treatment with benznidazole, we recorded clinical symptomatology and collected serum samples to run two ELISAs based on total and recombinant antigens in each yearly follow-up visit. Upon analyzing clinical and serological data, we found there were no major changes in the patients´ clinical status while serological reactivity generally declined until the third year of follow-up, particularly with the total antigen test. Remarkably, only one participant met the cure criterion of negative serological reversion for both ELISAs at the final visit. Our findings confirm that, using currently available tools, the appropriate time to address treatment efficacy is longer than five years. Additional studies are needed to find biomarkers for the early assessment of treatment efficacy.

## 1. Introduction

Chagas disease (CD) is a neglected tropical disease (NTD) caused by the parasite *Trypanosoma cruzi (T*. *cruzi)*. It is endemic to 21 countries in the Americas, where the disease exerts a major public health burden. According to World Health Organization (WHO) estimates, there are roughly seven million people infected [1], although this might be an underestimation considering that a large percentage of the people with the infection have not been diagnosed [2]. Bolivia has the highest prevalence of CD in the world, with an estimated 6.1% of its population infected [3]. Moreover, 60% of the country is endemic due to the reported presence of the vectors responsible for the main transmission route in seven of the country’s nine departments [4]. The magnitude of the disease impact comes with social, political, and economic consequences, including loss in revenue and workforce, loss in educational attainment, and an increase in healthcare spending. In fact, economic modeling has shown that identifying and treating at least 5% of acute and indeterminate CD cases each year could reduce transmission and provide economic and health protection [5].

The disease course begins with a mostly asymptomatic acute phase, lasting weeks to a few months. Then, it progresses to a long-lasting chronic phase in which clinical symptoms may appear ten to twenty years after the initial infection [6]. It is estimated that 20–30% of those chronically infected will develop life-compromising symptoms due to parasite-driven damage of cardiac and/or digestive tract tissues [6–8]. Diagnosis of the infection in the indeterminate (asymptomatic) chronic stage is based on the detection of specific antibodies against *T*. *cruzi* using serological tools such as immunofluorescence tests (IFIs), indirect hemagglutination (IHA) assays, or most commonly, enzyme-linked immunosorbent assays (ELISAs) [9–10]. Following current recommendations, due to the parasite antigenic diversity, diagnosis must be confirmed by at least two reactive serological tests based on distinct antigen sets [11].

After diagnosis is confirmed, there are two anti-parasitic drugs available to treat the infection: benznidazole (BNZ) and nifurtimox (NFX) [12]. These medications are highly efficacious when administered early upon infection, as observed in the case of congenitally infected newborns, who have high treatment tolerance [13]. However, their efficacy diminishes when provided to chronically infected adults, who have a lower tolerance for the drugs´ long administration regimens [12]. Moreover, complete negative seroconversion over time is the criterion of cure [14], but this can take many years to occur which makes it greatly impractical for disease monitoring [15].

Over the years, several studies have analyzed the co-evolving of clinical and serological parameters in chronically *T*. *cruzi-*infected subjects who received anti-parasitic treatment [16–18]. So far, despite the lack of tools to attain a timely assessment of treatment response, the beneficial effects of administering anti-parasitic drugs during the chronic stage of the infection are undoubtedly acknowledged [19–20]. Until much awaited tools for early treatment efficacy assessment are developed, the follow-up of treated patients must rely on current serological assays. With the aim to determine when, and how many, of such assays shall be used over time, we have evaluated the clinical evolution and serological status of a cohort of 106 chronically infected adults first attending to the Platform for Integral Care of Chagas disease Patients center (“the Platform”) [21] in the city of Cochabamba (Bolivia) between 2009–2013. We aim for these results to provide knowledge for improved CD case management, with reforms to follow-up plans for chronically infected patients.

## 2. Methods

### 2.1 Ethics statement

The study complied with the principles of the Declaration of Helsinki. The study protocol was reviewed and approved by the Ethical Committee of Fundación Ciencia y Estudios Aplicados para el Desarrollo en Salud y Medio Ambiente (CEADES) in Cochabamba, Bolivia. The approval reference code was CE-CH-S-290713. All participants included in the study signed an informed consent. For those under 18 years old, written formal consent was obtained from the parent/guardian and informed assent was provided by the minor.

### 2.2 Study design

A longitudinal observational study was conducted to collect data and samples from individuals attending the Platform in Cochabamba (Bolivia) between 2009 and 2018. Participation was offered on their first visit to those subjects ≥ 14 years old who attended the Platform in their first visit between 2009 and 2013 and were diagnosed for *T*. *cruzi* infection. All study participants were chronically infected and presented with indeterminate or symptomatic CD. Upon detection of the infection, and if the patient fulfilled criteria for chronic or indeterminate CD [22], administration of the standard BNZ dose (5 mg/kg/60 days) was provided to the participants. Due to ethical concerns concerning access to treatment, the study did not include a control arm. A baseline serum sample was taken before treatment (time zero) and then consecutively each year during routine examinations for a range of five years (year 1-year 5). During these yearly visits, clinical data was also captured, including electrocardiogram (ECG) results and additional cardiac and digestive symptoms. While all patients took the recommended dose of BNZ over the course of 60 days, there were differing start and end times among them.

All subjects in the study cohort were included upon confirmation of compliance with the inclusion criteria (≥ 15 years old and T. cruzi-infected). We excluded from the analysis those subjects who: (i) did not have a pre-treatment serum sample; (ii) had concomitant immunological compromise due to some underlying disease or the consumption of immunosuppressants; (iii) did not comply with the treatment regime; or (iv) underwent treatment in other services. Additional details of the study participants are in **Table 1**.

**Table 1:** Overview of study participants.

Participant Demographics (N = 106)	
Characteristics	Total Frequency (%)
**Sex**	
Female	72 (67.92)
Male	34 (32.08)
**Region**	
Rural	46 (43.40)
Urban	60 (56.60)
**Age at enrollment**	
14–30 yrs	18 (16.98)
31–40 yrs	24 (22.64)
41–50 yrs	34 (32.08)
51–62 yrs	30 (28.30)

### 2.3 Serological analysis

Whole blood samples were collected by venipuncture and left to coagulate following standard procedures. The obtained sera were aliquoted and stored at -20°C until needed. Serological diagnosis was performed according to currently recommended guidelines [11]. Two ELISAs based on the detection of IgG antibodies, one recombinant and one total antigen, were used to analyze each sample. These included the Wiener Chagatest Recombinant ELISA v3.0 based on recombinant antigens (Wiener Lab, Rosario, Argentina), and Lemos Chagatek ELISA based on whole lysate antigen (Laboratorios Lemos SRL, Buenos Aires, Argentina). For the baseline detection of subjects recruited in the early years, in addition to the total antigen ELISA, 47 samples were analyzed using IHA in place of the recombinant ELISA at baseline. All IHA samples had a positive readout. The use of IHA was due to the non-availability of the recombinant ELISA test during that early time of the study. After that, the same combination of ELISAs was used to test each patient’s samples throughout the course of the study. Assays were performed following the manufacturers´ instructions. Optical density (OD) readouts and cut-offs (which were used to determine a negative readout if the OD was below the cutoff) were recorded for each sample as well as the date of performance. In addition, after the initial ELISAs, all samples were re-assayed running together those from the same patient to address the potential influence of day-to-day variations in the performance of the techniques.

### 2.4 Clinical data collection

Clinical status of patients with *T*. *cruzi* infection was established based on signs/ symptoms of heart involvement (related to arrhythmia and heart failure), ECG and chest X-rays, following Kuschnir scale [22]. Annual ECGs were performed to compare changes between pre- and post-treatment periods. All ECGs were done with the same electrocardiograph and handled by highly trained Platform personnel. To conduct these ECG readings, a 12-lead tracing was taken with a 3-channel Schiller AT-1 or EDAN SE electrocardiograph. Patients were prepared in dorsal decubitus (lying on their back), with a 5-minute rest and free of objects that could cause interference. Verification of precordial and unipolar electrodes, evaluation of results, and their submission into the database was completed by the Platform’s care provider. Variables that were documented in the database included: the date and number of ECGs performed, presence or absence of cardiac symptomatology, and cardiomyopathy classification using the Kuschnir scale [22]. The recorded cardiac symptoms included typical chest pain, atypical chest pain, syncope, palpitations, edema, dyspnea, and lipothymia. In the absence of symptoms, the patient was registered as asymptomatic.

An annual medical check-up to collect information on any digestive symptoms the patient could be experiencing was also carried out during each ECG reading visit according to the Platform’s care protocols. Digestive symptoms included: dysphagia (liquid, solid), regurgitation, dyspepsia, constipation, abdominal distention and pyrosis (heartburn). The care provider also documented if the patient was asymptomatic or had any additional symptoms to report. After the medical exam, the provider compared the symptoms to the pre-treatment status and noted if there were no changes, progression, or regression. These changes were noted for the ECG, as well as for the registered cardiac and digestive symptoms.

### 2.5 Statistical analysis

The estimated sample size was calculated based on a population of inhabitants for Cochabamba and its peri-urban area, and a serological prevalence of 6.1% in adults [3]. Assuming that an estimated 20% have a seroconversion to negative eight to ten years afterwards, using a sample size of 106 participants, a 95% confidence interval can be estimated with a 7.6% precision.

Serological and clinical data were analyzed using STATA 17 (StataCorp.2021 Stata Statistical Software: Release 17. College Station, TX: StataCorp LLC) and Microsoft Excel. Data was reviewed to ensure all participants had, at the minimum, a baseline, midpoint, and endpoint serum sample as well as clinical data. Participants were excluded from the analysis if they did not include at least these three data points. Additionally, duplicate OD readouts for the same patient were dropped from the dataset. Descriptive statistics were used to summarize participant demographics and clinical data.

A longitudinal panel analysis was used to assess the serological evolution over time, which was selected to account for the heterogeneity between patient follow-up times and serological sample collections. OD readouts were plotted to visualize trends over time and determine when declines took place. Univariate logistic regressions were run to determine the strongest predictors of clinical changes (regression, no change, or progression). Wilcoxon rank-sum tests were performed to determine if there was a statistically significant difference between OD readouts at baseline and after each consecutive year of follow-up, with statistical significance set at *p* < 0.05 or 95% confidence interval (CI). Key tables and figures were created to demonstrate the clinical and serological evolution over the study period and its relation to treatment time.

## 3. Results

### 3.1 Participant demographics

The final study cohort included 106 participants, and 67.9% (n = 72/106) of these were female (**Table 1**). Participants had almost equal representation from urban and rural regions, with 56.6% (n = 60/106) living in an urban region and 43.4% (n = 46/106) living in a rural region. There was a wide age range of participants who enrolled in the study. The youngest participant who began treatment was 14 years old, while the oldest participant was 62 years old. The median age of participants enrolled was 44 years with a standard deviation of 10.7 years.

### 3.2 Clinical evolution

Numerous cardiac and digestive symptoms were documented during yearly follow-up visits with the Platform’s care provider (**Table 2**). While the ECG and chest X-ray readouts were measured by the care provider, the general cardiac and general digestive symptoms were self-reported by the patient. The ECG readouts showed that most patients (80.2%) had a normal electrocardiogram and no sign/ symptoms of heart enlargement during their final visit to the clinic after treatment. Additionally, these readouts showed that 16.0% of patients had an abnormal endpoint ECG, without signs/symptoms of heart enlargement. Another 3.8% of patients had visible heart enlargement in the baseline chest X-ray. Overall, over the course of the follow-up 94.3% (100/106) of participants had no changes in their ECG results when comparing to their baseline recordings, while 2.8% (3/106) had cardiomyopathy progression and other 2.8% (3/106) had regression over the course of the study, measured by ECG findings together with clinical symptoms of cardiomyopathy.

**Table 2:** Clinical outcomes at endpoint with baseline comparisons on: electrocardiogram readout, and reported general cardiac and digestive symptoms.

Category	Symptom or Outcome	Number of patients with symptom or outcome at final visit (%)
Electrocardiogram readout*	0	85 (80.19)
	I	17 (16.04)
	II	4 (3.77)
*Changes in baseline cardiomyopathy*	***No changes***	100 (94.34)
	***Progression***	3 (2.83)
	***Regression***	3 (2.83)
General cardiac	Asymptomatic cardiac presentation	54 (51.43)
	Typical chest pain	0 (0.00)
	Atypical chest pain	46 (43.40)
	Syncope	9 (8.49)
	Palpitations	12 (11.32)
	Edema	0 (0.00)
	Dyspnea	1 (0.94)
	Lipothymia	0 (0.00)
	Other cardiac symptoms (uncategorized)	1 (0.94)
General digestive	Asymptomatic digestive presentation	60 (56.60)
	Solid dysphagia	6 (5.66)
	Liquid dysphagia	1 (0.94)
	Regurgitation	3 (2.83)
	Dyspepsia	35 (33.33)
	Constipation	4 (3.77)
	Abnormal distention	17 (16.04)
	Pyrosis (heartburn)	12 (11.32)
	Other digestive symptoms (uncategorized)	0 (0.00)
*Changes in baseline general cardiac and/or digestive symptoms*	***No changes***	41 (38.68)
	***Progression***	3 (2.83)
	***Regression***	62 (58.49)

*Modified Kuschnir classification of Chagas cardiomyopathy: 0, normal ECG findings and normal heart size (usually based on chest radiography, signs/ symptoms of left ventricular (LV) enlargement and heart failure); I, abnormal ECG findings without signs/ symptoms of LV enlargement and heart failure); II, LV enlargement; III, congestive heart failure.

For general symptoms, the patient cohort had more self-reported digestive symptoms than cardiac symptoms. For cardiac symptoms, the highest reported were atypical chest pain (46/106; 43.4%), heart palpitations (12/106; 11.3%), and syncope (9/106; 8.5%). For digestive symptoms, the highest reported were dyspepsia (35/106; 33.3%), abnormal distention (17/106; 16.0%), and heartburn (12/106; 11.3%). Interestingly, only 38.7% (41/106) of participants had no changes in their reported general cardiac or digestive symptoms compared to their baseline report. While 2.8% (3/106) of participants had a progression in their symptomatology, the majority (58.5%; 62/106) had a regression in these general symptoms. Among the three participants that had cardiac progression compared to their baseline cardiomyopathy readout, no participants self-reported cardiac or digestive symptoms. Among the three participants that had improvements in their cardiomyopathy compared to baseline, no participants had self-reported digestive symptoms. Additionally, one participant also had a regression in their baseline cardio and digestive symptoms while the other two participants had no changes.

### 3.3 Serological evolution over the 5-year follow-up period

#### 3.3.1 Change overview

**Fig 1:**
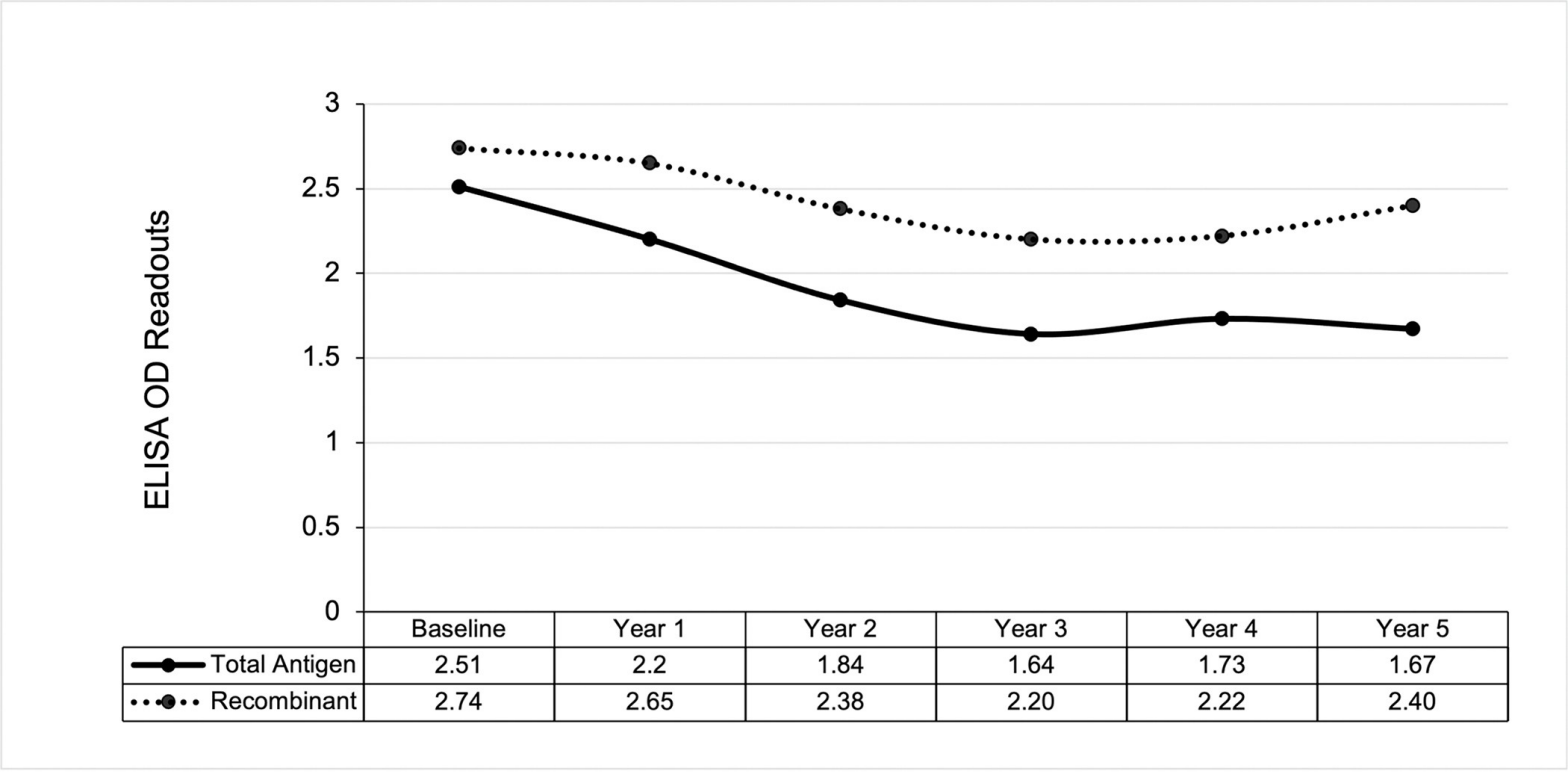
Average OD decline over five years. The average OD declined for both test types from baseline to year five. The total antigen ELISA is represented by a solid line; the recombinant ELISA is represented by a dotted line.

Serological readouts from both ELISA types demonstrated a decline in OD readouts from baseline to year 3 recorded signals with a small increase observed in average OD results during the final two years of follow-up (years 4 and 5) (**Fig 1**). Notably, while the total antigen ELISAs had an average net OD decrease of 0.84, the recombinant ELISAs had an average net OD decrease of 0.34. Both tests had the lowest average OD readouts in year 3.

**Fig 2:**
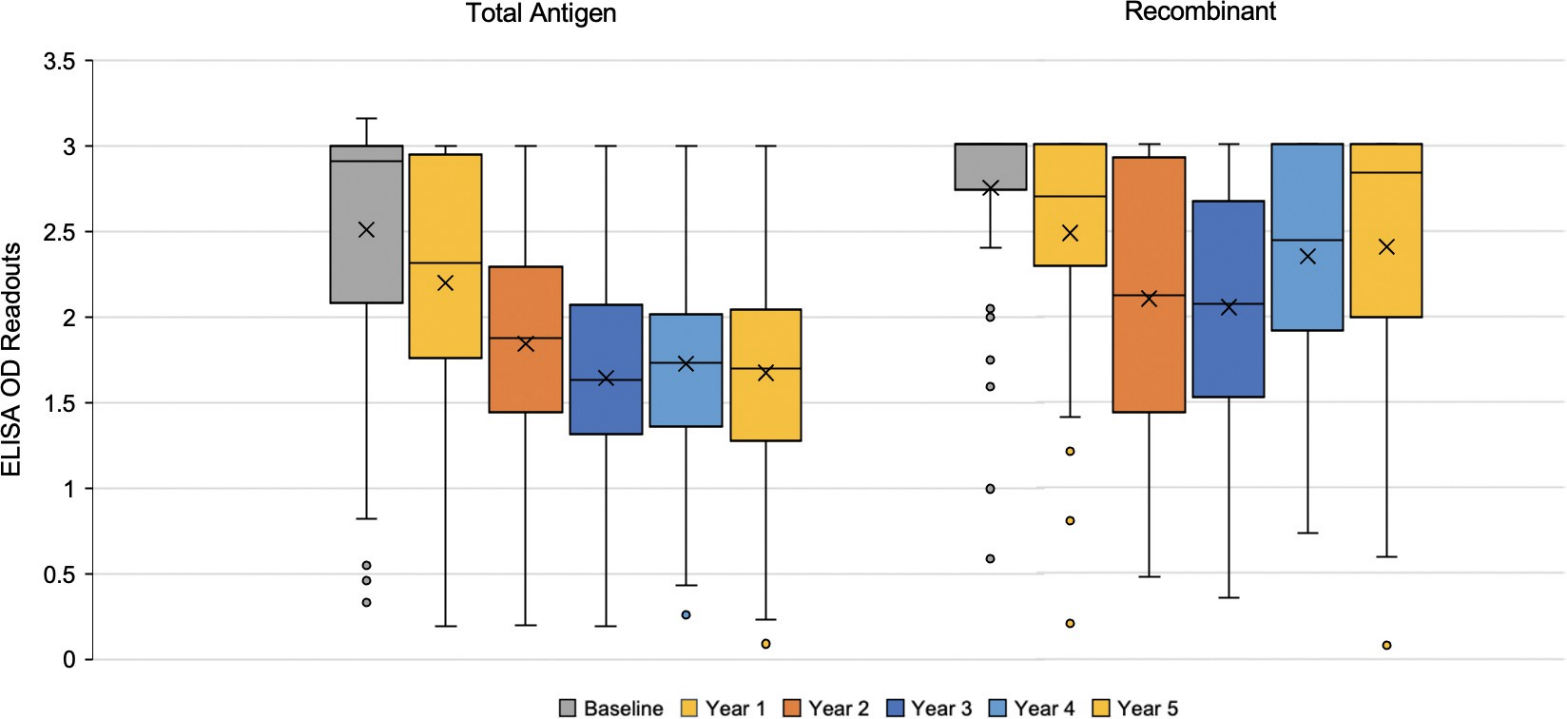
Five-year evolution of OD readouts from ELISA tests based on total antigen and recombinant antigen. Box plots were used to graph the variation in OD readouts among the study sample for each year (total antigen- left; recombinant- right).

Box and whisker plots of the OD readouts show similar trends for each ELISA test type. For the total antigen ELISA results, the median OD was the lowest in year 3 (**Fig 2**). As the time progressed, the upper and lower quartiles decreased. Compared to the total antigen readouts, the recombinant readouts showed less consistency over the years. Like the total antigen readouts, the median OD for the recombinant readouts was also the lowest in year 3 (**Fig 2**), although the medians for years 4 and 5 were much higher compared to the total antigen ELISA. Additionally, there were more outliers in the recombinant ELISA readouts for baseline and year 1 data compared to those of the total antigen test. Wilcoxon rank-sum tests showed that there was a highly significant difference (*p* < 0.001) between the baseline OD and each year of follow-up for both ELISA assays used (see **S1 and S2 Figs,** respectively for total and recombinant antigen ELISAs).

#### 3.3.2 Serological evolution—percent change over time

**Fig 3:**
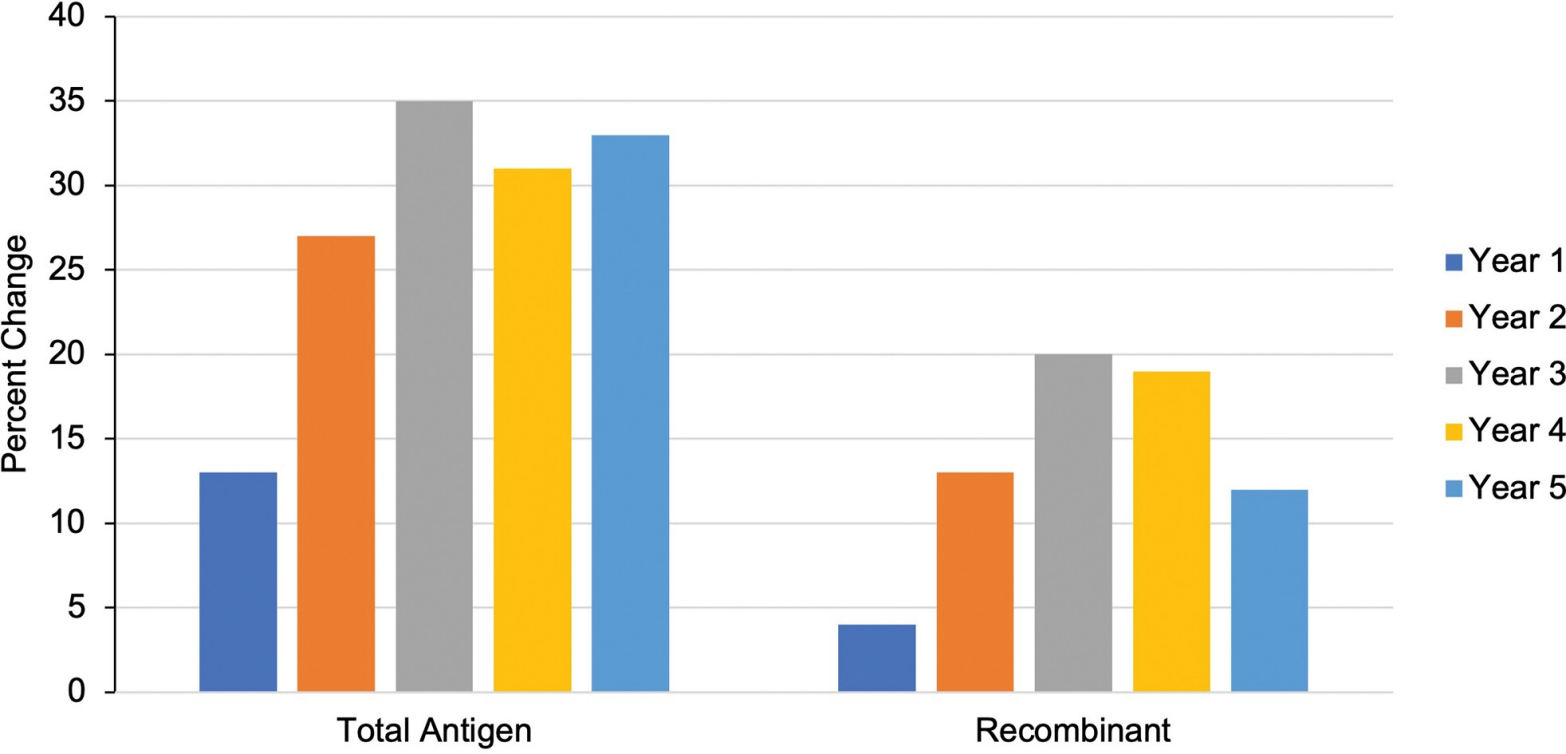
OD percent change. Percent change from baseline was calculated for each year by test type (total antigen- left; recombinant- right).

By analyzing the percent change of the OD readouts, additional information on the serological evolution of readouts was gained (**S1 Table**). Overall, only one participant tested negative (had an OD readout less than the provided cutoff to determine positivity) for both ELISA types during the final year and could thus be informed as cured (**S3 and S4 Figs**). We also compared the OD percent change from baseline to each year of follow-up depending on the ELISA test type (**Fig 3**). Results retrieved with the total antigen ELISA experienced a greater percent change for each year compared to the recombinant ELISA readouts. Its greatest percent change to baseline occurred in year 3, with a net percent change of 34.5% (**Fig 3**). Similarly, the recombinant ELISA results had the greatest percent change in year 3 with a net change of 19.6% (**Fig 3**).

**Fig 4:**
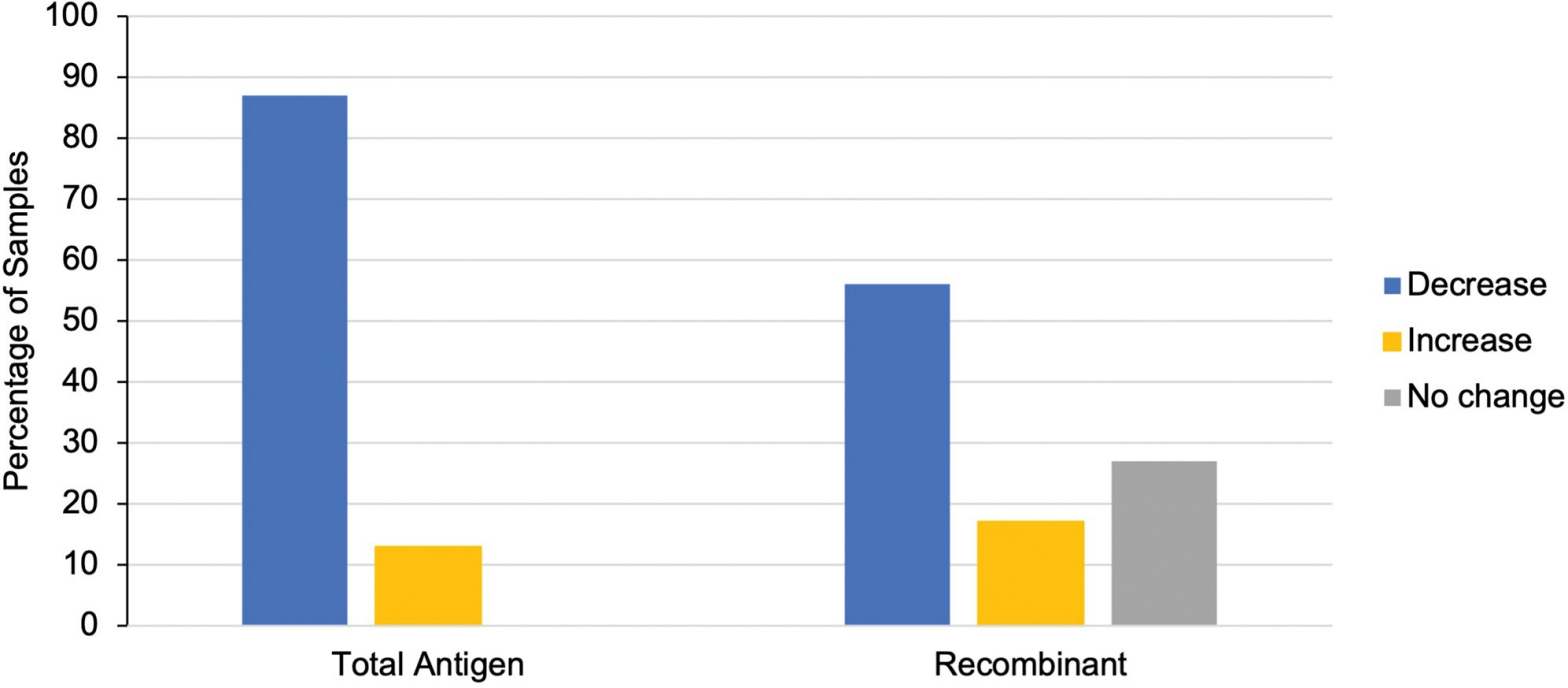
OD change from baseline to final year. This graph presents the percentage of samples that had either a 1) net increase, 2) net decrease, or 3) no change in their OD readouts from baseline to year five (total antigen- left; recombinant- right).

The net OD change (baseline to final year of follow-up) was also compared by test type (**Fig 4**). Results showed that while 87% of participants (n = 92/106) had a net OD decrease in their total antigen ELISA, only 56% (n = 33/59) had a net decrease in the recombinant ELISA (**Fig 4**). A similar number of participants had a net OD increase in both test types, with 13% (n = 14/106) and 17% (n = 10/59) of total antigen and recombinant readouts, respectively. Unlike the total antigen readouts, 27% (n = 16/59) of participants had no net change at all in their recombinant readouts.

While there were differences in the ELISA OD readouts between the two test types, the test results (positive or negative) were very similar for each year. Out of the 530 sets of tests conducted between the five years, 99.1% (525/530) had matched results with mismatches occurring in years 1 and 3 only. For example, in year 1, one participant had a negative total antigen readout with a positive recombinant readout. A second participant had opposite results (a positive total antigen readout with a negative recombinant readout). In year 3, two participants had a positive total antigen readout with indeterminant recombinant readouts. A third participant had a negative total antigen readout with a positive recombinant readout.

**Fig 5:**
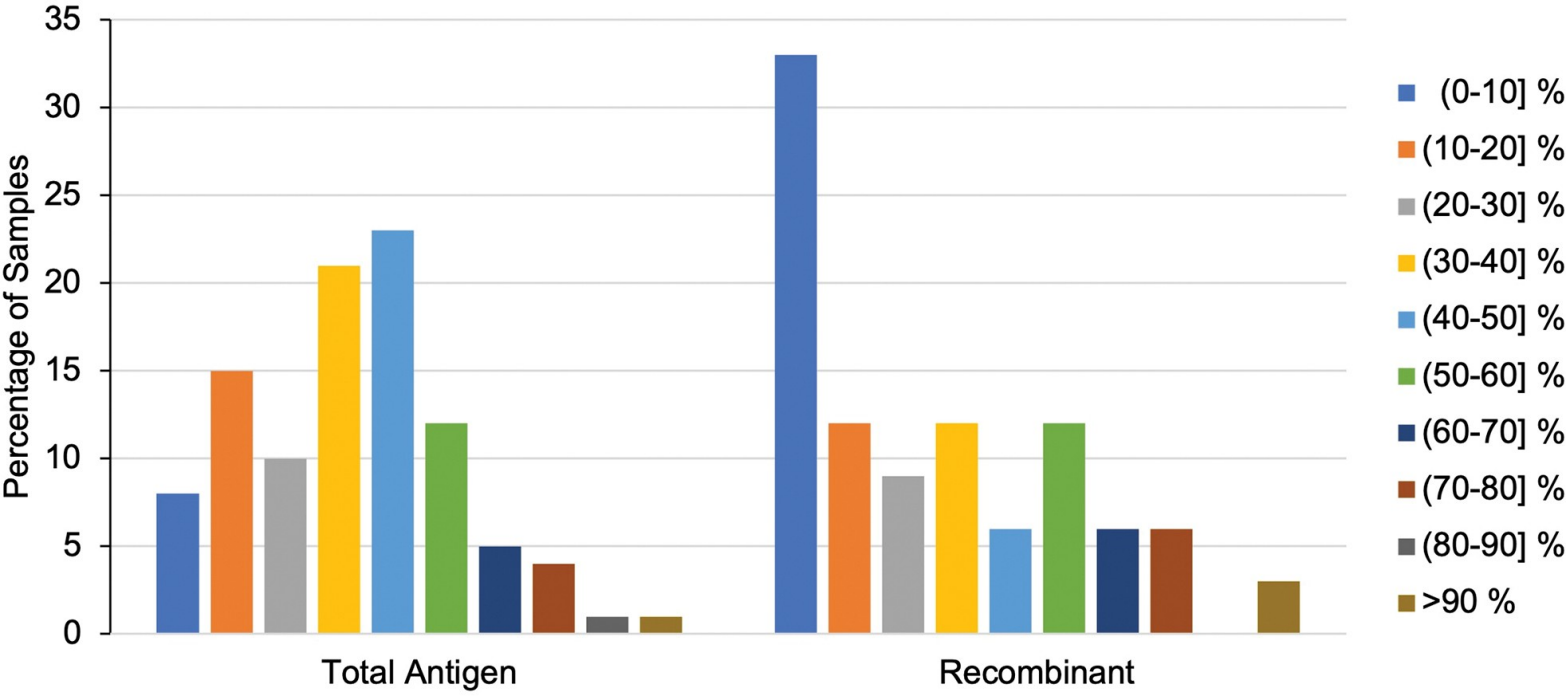
Ranked OD net percent decrease. This graph presents the net decrease of samples by test type (total antigen- left; recombinant- right).

Lastly, the changes observed in OD values recorded for all samples from baseline to the final year of follow-up (year 5) were grouped by net decrease range to better understand those variations by test type in a more quantitative manner (**Fig 5**). We found that total antigen ELISA readouts showed that the largest number of participants experienced a net decline (percent change from baseline to year 5) in the range of 40–50% (**Fig 5**). In contrast, the readouts retrieved with the recombinant ELISA showed that the largest number of participants experienced a 0–10% decline (**Fig 5**). Very few participants experienced net declines greater than 60% with either test.

### 3.4 Relationship between clinical and serological evolution

A univariate logistic regression found that females who completed BNZ treatment were 3.8 times more likely to have a normal ECG readout during their final clinic visit compared to males who completed the same treatment (**Table 3**). Similarly, a second logistic regression showed that females who completed BNZ treatment were 2.4 times more likely to have a regression in their general cardiac and digestive symptoms by their final clinic visit compared to males who completed the same treatment (**Table 4**). No significant relationship was identified when analyzing the independent variables of age, location, and decrease in total antigen or recombinant OD readouts.

**Table 3:** Univariate logistic regression for predictors of normal ECG readout (Kuschnir = 0)* compared to abnormal ECG readout.

Univariate logistic regression for predictors of “normal ECG readout”	Normal ECG readout at final clinic visit (N = 85/106)					
Characteristics	Frequency	%	Odds Ratio	Lower Limit	Upper Limit	*p*-value
**Sex (ref: male)**						
Female	63	74.12	3.82	1.42	10.28	0.01
**Age (ref: 14–30 yrs)**						
31–40 yrs	17	20.0	0.14	0.02	1.29	0.08
41–50 yrs	24	28.24	0.14	0.02	1.21	0.07
51–62 yrs	27	31.76	0.63	0.51	5.51	0.60
**Location (ref: urban)**						
Rural	40	89.96	2.22	0.79	6.28	0.13
**Total Antigen OD Readouts (ref: increase)**						
Decrease	76	82.61	2.64	0.78	8.93	0.12
**Recombinant OD Readouts (ref: increase)**						
Decrease	26	55.32	0.41	0.04	3.83	0.44
No change	12	25.53	0.33	0.03	3.52	0.36

*Modified Kuschnir classification of Chagas cardiomyopathy: 0, normal ECG findings and normal heart size (usually based on chest radiography, sign/ symptoms of left ventricular (LV) enlargement and heart failure).

**Table 4:** Univariate logistic regression for predictors of general symptoms regression compared to no change or symptom progression.

Univariate logistic regression for predictors of Normal ECG Readout	General Cardiac or Digestive (Regression) (N = 62/106)					
Characteristics	Frequency	%	OR	Lower Limit	Upper Limit	p-value
**Sex (ref: male)**						
Female	47	75.81	2.38	1.04	5.48	0.04
**Age (ref: 14–30 yrs)**						
31–40 yrs	16	25.81	2.00	0.57	7.01	0.28
41–50 yrs	20	32.26	1.43	0.45	4.51	0.54
51–62 yrs	17	27.42	1.31	0.41	4.23	0.65
**Location (ref: urban)**						
Rural	30	48.39	1.64	0.74	3.62	0.22
**Total Antigen OD Readouts (ref: increase)**						
Decrease	54	87.10	1.07	0.34	3.32	0.91
**Recombinant OD Readouts (ref: increase)**						
Decrease	17	54.84	2.48	0.55	11.28	0.24
No change	11	35.48	5.13	0.92	28.57	0.06

## 4. Discussion

Analyzing the serological evolution of CD, as well as changes to clinical symptoms, is necessary to understand the current limitations of existing diagnostics, treatments, and care management practices. In this study, we evaluated the serological progression of 106 chronically *T*. *cruzi-*infected participants over a five-year period after they completed the recommended course of BNZ, focusing on changes to OD readouts (increase/ decrease/ no change) between total antigen and recombinant antigen ELISA tests. Additionally, we measured and compared endline and baseline clinical outcomes of patients treated for CD and analyzed their relationship between demographic and serological characteristics. Our results showed that while only one participant met the “cure criterion” of a negative serological readout for both ELISAs by the final year of follow-up, most participants experienced OD declines associated with no changes or improvements to clinical symptoms compared to baseline.

Clinical outcomes measured throughout the study showed that most participants (80.2%) had a normal ECG readout during the final visit and most (94.3%) had no changes to their baseline cardiomyopathy, leaving only a few participants that experienced physician-measured symptom progression after taking BNZ. On the other hand, participants self-reported numerous cardiac and digestive symptoms, in which it is not clear if these symptoms are from the disease itself, or potential comorbidities, including non-organic symptomatology.

The efficacy of BNZ as a medication to treat CD has been assessed by many, with ranges of 14.3–44.6% efficacy for chronic patients [23], and 50–80% for acute cases [24–25]. A retrospective cohort study conducted by Hasslocher-Moreno found that BNZ treatment was associated with a decreased incidence of CD progression from indeterminate to chronic (with cardiac symptoms), with a cumulative incidence of 7.9% in treated patients compared to 21.1% in non-treated patients over a median follow-up of 15.1 years [26]. Furthermore, a study by Fragata-Filho et al. also reported that patients with normal ECGs at their first visit who received BNZ treatment had, over a 20-year period, lower prevalence of ECG abnormalities and relevant clinical events, such as heart failure, stroke, and mortality [18]. These findings underscore the benefits of BNZ treatment in maintaining normal ECG patterns and preventing significant clinical events. Similarly, a systematic review and meta-analysis by Perez-Molina and co-workers found that there was a significant increase in the probability of a response to BNZ therapy compared to placebo or no treatment, particularly in clinical trials [27]. Although the authors noted that the efficacy in the late chronic phase was not as supported, this uncertainty was attributed to many factors, including variation in the study population and differing endpoints and follow-up periods. The analysis highlighted that BNZ treatment significantly reduced the risk of clinical events among CD patients. Overall, evidence suggests that the administration of anti-parasitic treatment is effective in preventing CD progression from indeterminate to chronic, maintaining normal ECG patterns, and greatly reducing the risk of clinical events among patients with normal ECGs. However, to better address the effects of BNZ, it is important for care providers to document the serological and clinical evolution with larger, randomized cohorts and with longer follow-up periods to provide a more conclusive understanding of changes and their relation to CD.

In this study, logistic regression analyses demonstrated that females were more likely to have a normal ECG readout by year 5 (endline) as well as improvements in their cardiac and digestive symptoms after taking BNZ compared to male participants. While this finding may be linked to gender differences in medical care service utilization and healthcare information seeking behaviors, such as presented by Ek [28] and Bertakis and coworkers [29], additional studies should be performed to understand how BNZ efficacy and self-reported symptomatology may differ based on sex. Nonetheless, barriers to CD screening and care are still prominent, including lack of knowledge of the disease among healthcare providers [28], low funding, and limitations in accessible diagnostics [30–31,3]. To increase access to care and awareness of CD in endemic countries, one example was the implementation, together with local health authorities of the Bolivian Platform for the comprehensive care of adults with CD in 2009, with efforts to provide care, train health professionals, and expand the model to the national health system of Bolivia and to other endemic countries [21].

The total antigen and recombinant ELISA readouts provided key insights into OD patterns over the five-year period as well as differences in test type results. To begin, both ELISAs documented average declines up to year 3 and slight inclines for the following two years. This observation suggests that chronic CD patients should be followed by providers for a longer period to document additional serological and clinical changes, especially as near 100% of participants in our study were not cured by year 5, as reported in previous observations [32–34]. Additionally, these changes in OD values raise awareness to the effectiveness of this medication, as previously discussed.

Results also showed clear differences between the total antigen and recombinant ELISAs, although both tests followed similar patterns of decline and incline throughout the study. For example, the total antigen ELISA readouts showed less variation in their average OD decline and more participants with a net decrease in their readouts. The recombinant ELISA readouts, on the other hand, showed larger variation in average decline, fewer participants with a net OD decrease, and documented participants who experienced “no change” in their OD readouts compared to baseline. Furthermore, the current approach for diagnosing CD requires two tests, either that use different techniques or detect antibodies to different antigens [35]. Additional studies should investigate the necessity of this approach as well as the optimal test to be used, as it is possible that accurate CD diagnoses can be made based on the reliance of a single ELISA test, which has many downstream benefits for testing in low-resource settings. Additional studies are also investigating alternative ELISAs, like next-generation assays based on the combination of short peptidic epitopes [36]. Nevertheless, in what respects to the use of paired tests for the follow-up, attending to a 100% agreement between total antigen and recombinant ELISAs observed in this work, the use of a single assay shall be suggested. Moreover, following the results retrieved here in Cochabamba, such a test should be the total antigen one.

This study had multiple limitations. First, a control group was not included due to the ethical concerns of withholding treatment for patients who tested positive for CD. Therefore, we could not draw conclusions about clinical or serological outcomes compared to a cohort of untreated participants in this time frame. Furthermore, the serological analysis of the samples by ELISA during this multi-year study was done at distinct timings by several laboratory technicians, which may have led to variations in terms of sample management, storage or assay performance. Notably, to confirm the accuracy of the results, samples from all participants were run on fresh assays by the same lab technician and then compared to the original readouts, which served as an important sensitivity analysis and confirmed the accuracy of the original readouts. On the other side, the clinical data captured in the study also presented limitations in the analysis. Mainly, that the endline general cardiac and digestive symptoms documented in the study were self-reported by the patients. Therefore, the presence of self-reporting biases, such as social desirability or recall bias cannot be discarded. Anyhow, additional physician-measured symptoms (including ECG and change variables) were collected throughout the study to provide a more objective measurement for the analysis.

## 5. Conclusions

The primary aim of this study was to detect the serological changes in Bolivian patients five years after finishing anti-parasitic treatment with BNZ, while also looking at changes to clinical status over the same timeframe. The results show that while one participant became “cured” of CD by the final follow-up (year 5), most participants still had a decline in their positive OD readouts after treatment. The recorded clinical parameters showed that most patients did not have any significant changes to their cardiac or digestive symptoms after treatment, at least in the time frame under investigation, while a small percentage demonstrated either a regression or progression in symptoms. Overall, these findings suggest that the appropriate years of patient follow-up to determine if patients treated with BNZ have been cured of CD is longer than five years. In terms of serological reactivity evolution, we found that using the total antigen ELISA might be a more reliable measure, at least in the region of Bolivia where the study was developed. To provide more equitable and effective care to Chagas disease patients, further work must be done to develop more effective CD diagnostics, treatments, response to treatment and progression markers, and care management protocols, especially for use in low-resource settings where people are most affected.

## Supporting information

S1 Table Summary table of serological evolution sample characteristics. (DOCX)

S2 Table Table with all data used to draw the conclusions outlined in the manuscript in agreement with PLOS´ data policy. (XLSX)

S1 Fig Year-by-year comparison of OD readouts for total antigen ELISA. All comparisons were found to be statistically significant (*p* < 0.01).
(TIF)

S2 Fig Year-by-year comparison of OD readouts for recombinant ELISA. All comparisons were found to be statistically significant (*p* < 0.01).
(TIF)

S3 Fig Percentage of positive OD readouts. This figure presents the percentage of samples with a positive OD readout for each year (total antigen- left; recombinant- right).
(TIF)

S4 Fig ELISA OD readouts from one participant who reached the cure criterion. According to current guidelines, cure was defined by having negative serological readouts from both tests by the final year of follow-up (year 5).
(TIF)
