# Supplementary material for: Five-year serological and clinical evolution of chronic Chagas disease patients in Cochabamba, Bolivia
Source: PLoS Negl Trop Dis. 2023 Dec 29;17(12):e0011498. doi: 10.1371/journal.pntd.0011498 (PMC10756508; doi:10.1371/journal.pntd.0011498)
Supplement: S1 Table — (DOCX) [file pntd.0011498.s001.docx]

**S1_Table. Summary table of serological evolution sample characteristics.**

| Characteristic | Variable | **Summary Statistics** | | | |
| --- | --- | --- | --- | --- | --- |
|  |  | **Total Antigen** | | **Recombinant** | |
| **Positive readouts** | Baseline | 106 | 100% | 59* | 100% |
|  | Year 1 | 105 | 99% | 105 | 99% |
|  | Year 2 | 106 | 100% | 106 | 100% |
|  | Year 3 | 105 | 99% | 104 | 98% |
|  | Year 4 | 106 | 100% | 106 | 100% |
|  | Year 5 | 105 | 99% | 105 | 99% |
| **Percent change from baseline** | Baseline | 2.51 | - | 2.74 | - |
|  | Year 1 | 2.20 | 12.5% | 2.65 | 3.6% |
|  | Year 2 | 1.84 | 26.6% | 2.38 | 13.1% |
|  | Year 3 | 1.64 | 34.5% | 2.20 | 19.6% |
|  | Year 4 | 1.73 | 31.2% | 2.22 | 19.1% |
|  | Year 5 | 1.67 | 33.3% | 2.40 | 12.3% |
| **Net variation** | Decrease | 92 | 87% | 33 | 56% |
|  | Increase | 14 | 13% | 10 | 17% |
|  | No change | 0 | 0% | 16 | 27% |
|  | **Total** | **106** | **100%** | **59** | **100%** |
| **Percent decrease** | (0-10] % | 7 | 8% | 11 | 33% |
|  | (10-20] % | 14 | 15% | 4 | 12% |
|  | (20-30] % | 9 | 10% | 3 | 9% |
|  | (30-40] % | 19 | 21% | 4 | 12% |
|  | (40-50] % | 21 | 23% | 2 | 6% |
|  | (50-60] % | 11 | 12% | 4 | 12% |
|  | (60-70] % | 5 | 5% | 2 | 6% |
|  | (70-80] % | 4 | 4% | 2 | 6% |
|  | (80-90] % | 1 | 1% | 0 | 0% |
|  | >90% | 1 | 1% | 1 | 3% |

* 47 samples were analyzed using IHA in place of a recombinant ELISA during baseline; All IHA samples had a positive readout.
