# Supplementary figures and images for: Five-year serological and clinical evolution of chronic Chagas disease patients in Cochabamba, Bolivia
Source: PLoS Negl Trop Dis. 2023 Dec 29;17(12):e0011498. doi: 10.1371/journal.pntd.0011498 (PMC10756508; doi:10.1371/journal.pntd.0011498)

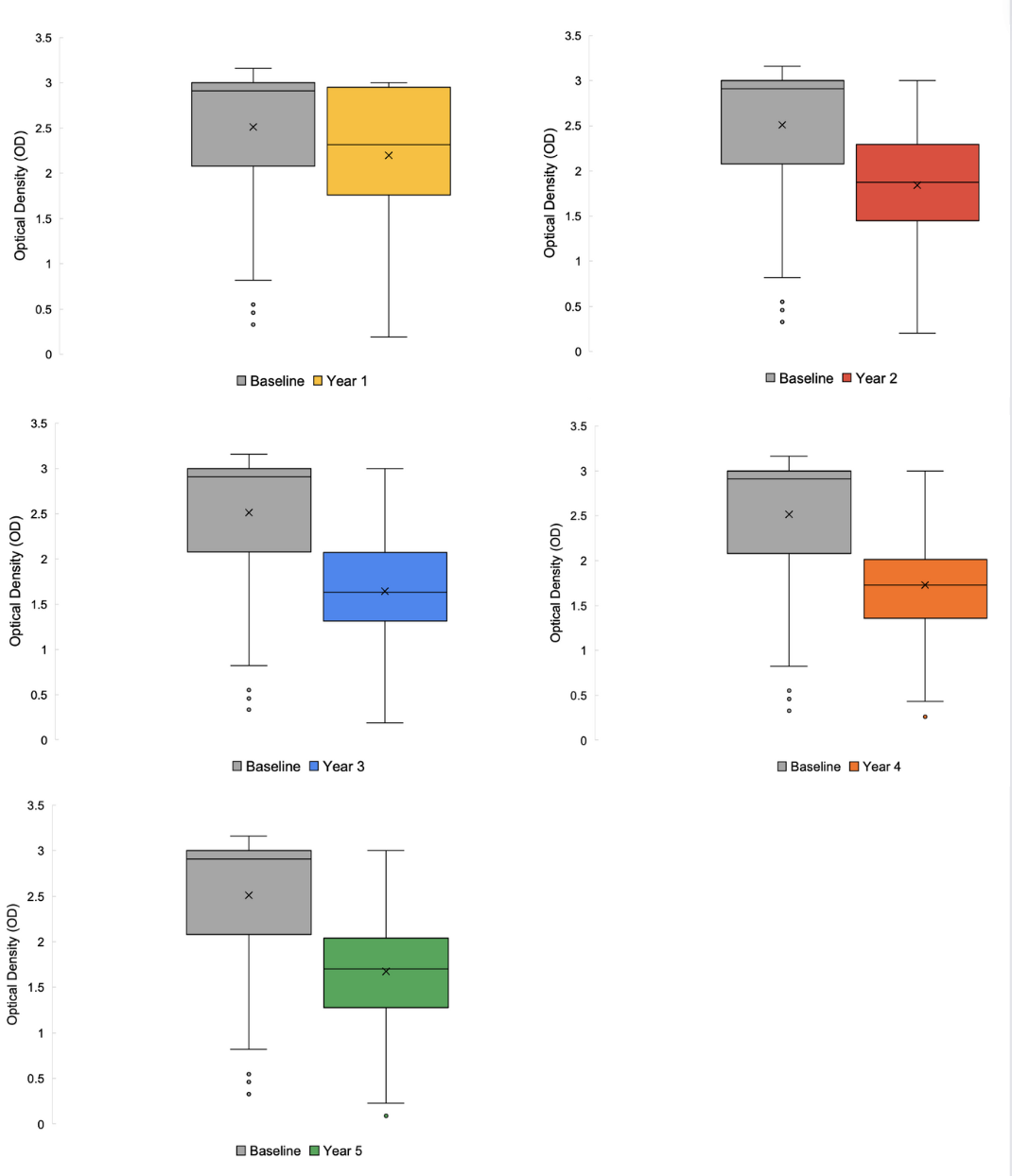

Supplement: S1 Fig — All comparisons were found to be statistically significant (p < 0.01). (TIF) [file pntd.0011498.s003.tif]

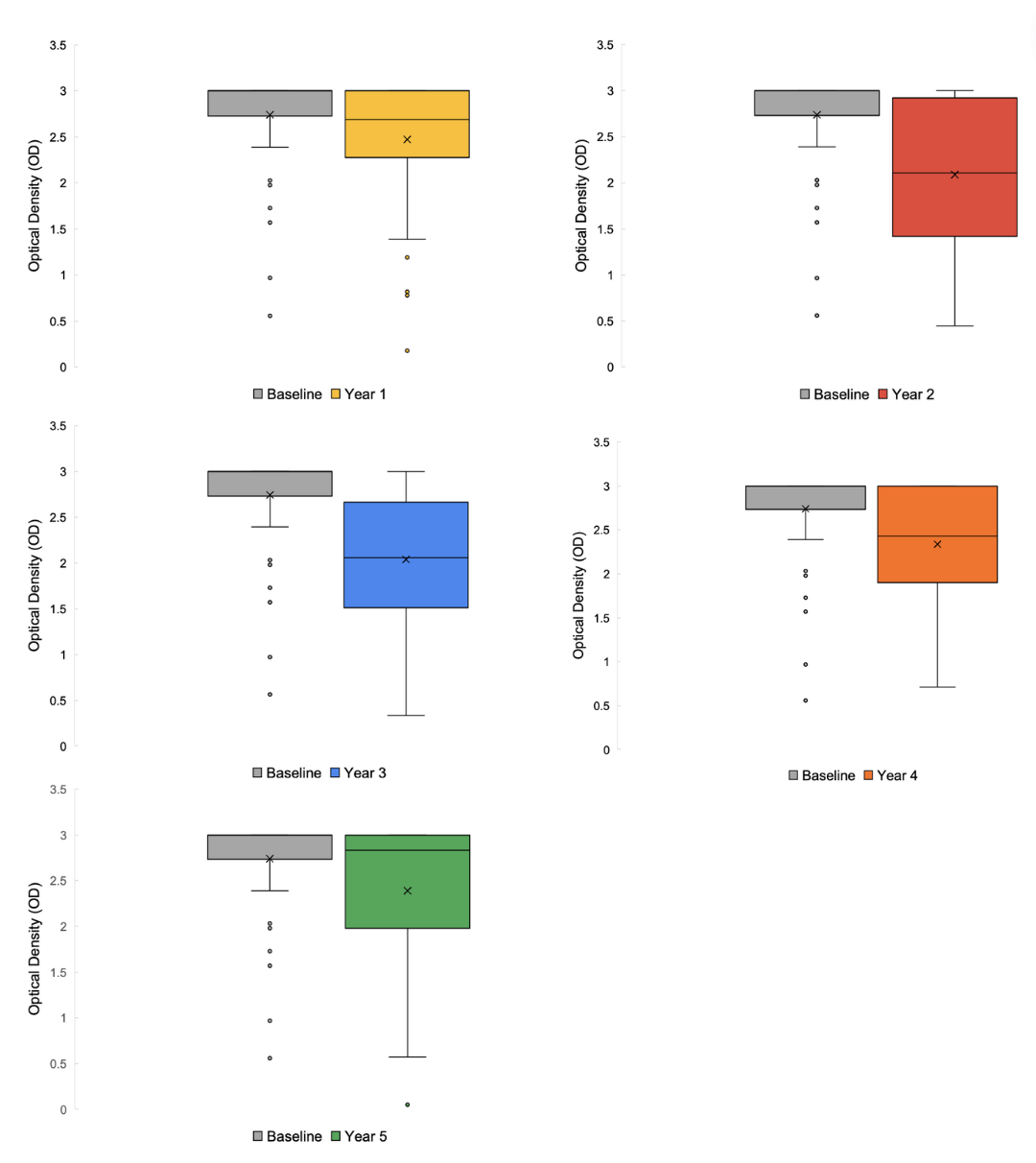

Supplement: S2 Fig — All comparisons were found to be statistically significant (p < 0.01). (TIF) [file pntd.0011498.s004.tif]

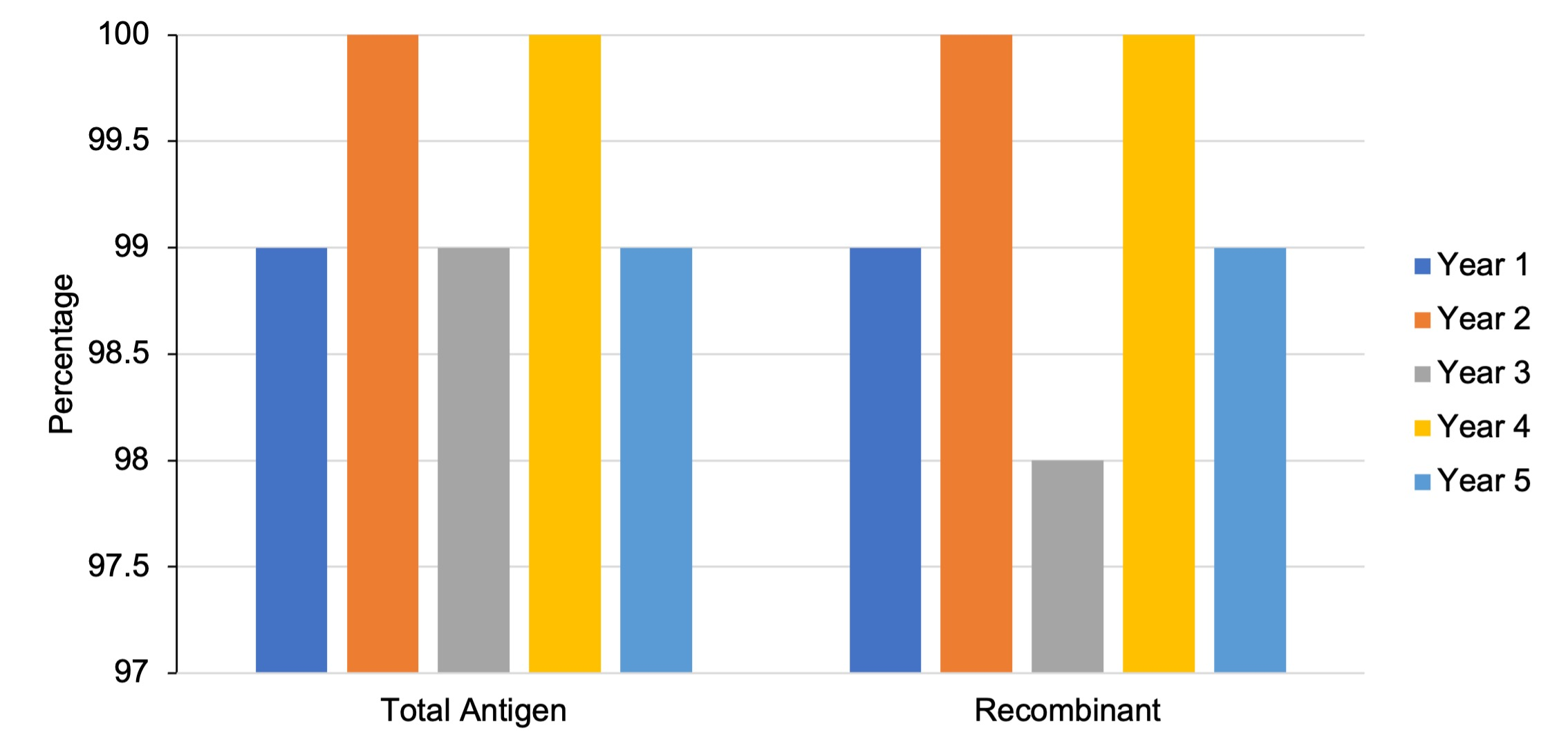

Supplement: S3 Fig — This figure presents the percentage of samples with a positive OD readout for each year (total antigen- left; recombinant- right). (TIF) [file pntd.0011498.s005.tif]

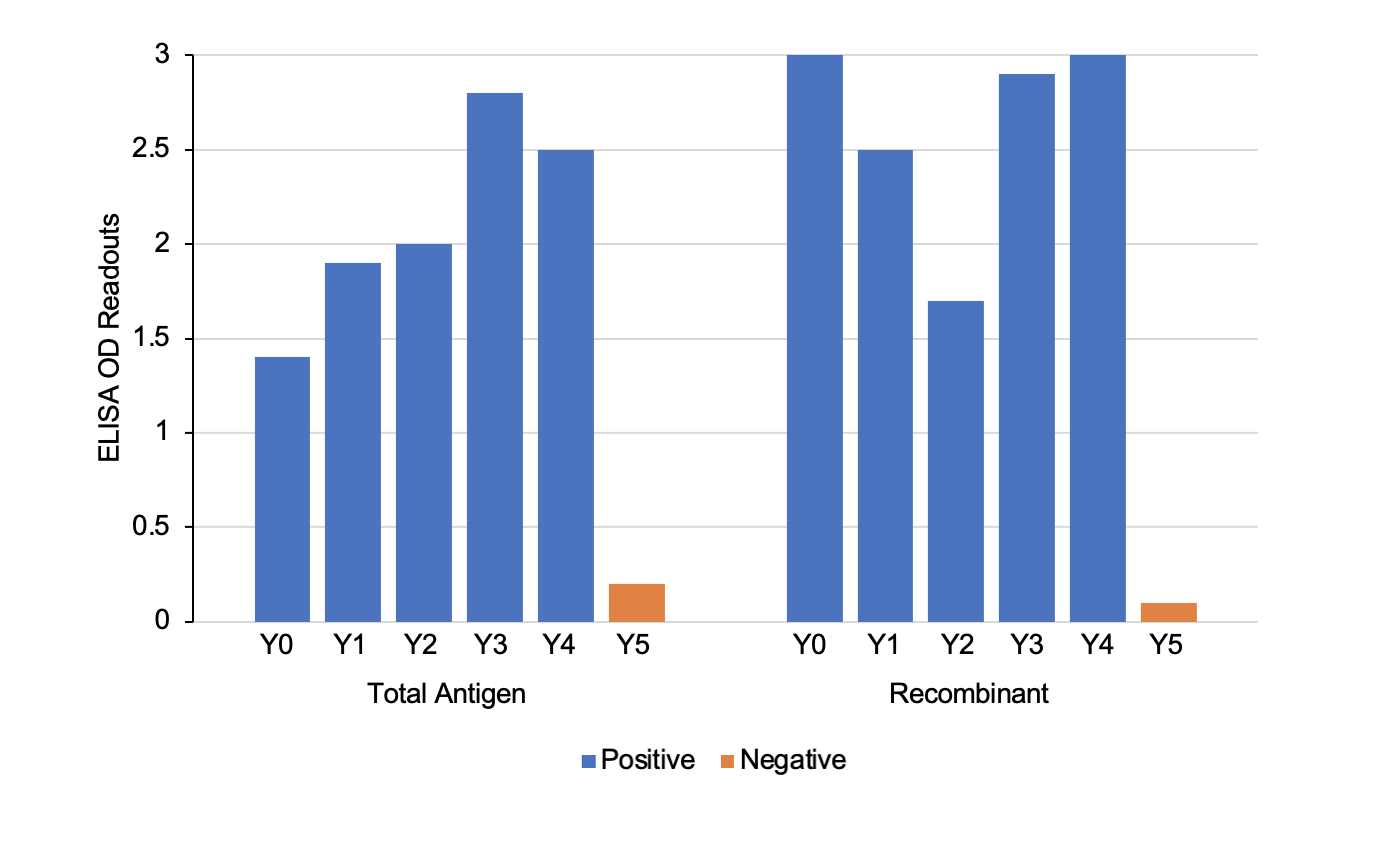

Supplement: S4 Fig — According to current guidelines, cure was defined by having negative serological readouts from both tests by the final year of follow-up (year 5). (TIF) [file pntd.0011498.s006.tif]
